# Functional Rehabilitation for Medial Gastrocnemius Silent Contractures to Prevent Foot and Ankle Disorders: A Review

**DOI:** 10.3390/muscles3040028

**Published:** 2024-09-25

**Authors:** Rafael A. Bernardes, Vítor Parola, Arménio Cruz, Nuno Correia, Hugo Neves

**Affiliations:** 1 Centre for Interdisciplinary Research in Health, Faculty of Health Sciences and Nursing, Universidade Católica Portuguesa, 1649-023 Lisbon, Portugal; rbernardes@ucp.pt; 2 The Health Sciences Research Unit: Nursing (UICISA: E), Nursing School of Coimbra (ESEnfC), 3000-232 Coimbra, Portugal; vitorparola@esenfc.pt (V.P.); acruz@esenfc.pt (A.C.); 3 ciTechCare, School of Health Sciences, Polythecni c of Leiria, 2411-901 Leiria, Portugal; nuno.correia@ipleiria.pt

**Keywords:** foot and ankle disorders, gastrocnemius contracture, rehabilitation nursing, functional rehabilitation, trauma care

## Abstract

Medial gastrocnemius silent contractures (MGSCs) are prevalent, notably impacting functional status and increasing the risk of foot and ankle disorders, especially among aging populations. Although traditionally managed by podiatrists and physiotherapists, the role of rehabilitation nursing in addressing MGSCs is gaining recognition. This paper elucidates the contributions of rehabilitation nursing to the functional rehabilitation of MGSC patients and underscores its vital role within the multidisciplinary team. Initially, the paper defines the clinical and physiological characteristics of MGSCs and their implications in foot and ankle disorders. It then meticulously explores rehabilitation nursing interventions—including personalized stretching regimens, vibration therapy, balance exercises, and judicious footwear selection—emphasizing their efficacy in enhancing muscle flexibility, joint mobility, and postural stability. The emphasis is on patient-centered approaches and education to foster treatment adherence and positive rehabilitation outcomes. The significance of interdisciplinary collaboration is highlighted, focusing on how rehabilitation nursing optimizes patient care and mitigates complications. The paper advocates for recognizing and integrating rehabilitation nursing in managing MGSC-related disorders, emphasizing its importance in achieving successful functional outcomes.

## 1. Introduction

Foot and ankle disorders prevail across various demographics, notably among professionals such as nurses who endure prolonged standing and walking [1], and in older adults where the incidence of sarcopenia augments susceptibility [2]. Remarkably, the prevalence of these disorders in the elderly can escalate to a significant 65%, often correlating with fall incidents [3,4]. The subsequent impairment of functional status and mobility underscores the severity of these conditions [5].

Within this spectrum, traumatic disorders like fractures become a common aftermath of falls in older adults. Recent studies delineate nonspecific disorders, including hallux valgus and Taylor bunion, as pivotal stability factors influencing the frequency of falls within this demographic [6]. The spectrum also encompasses nontraumatic disorders such as plantar fasciitis and Achilles tendinitis, often resulting from untreated calf syndromes [7]. As emphasized by Amis and colleagues, silent gastrocnemius contractures inflict gradual, significant damage to the foot and ankle, particularly when preventive measures are overlooked.

Delving into the anatomy, the gastrocnemius and the soleus—the posterior compartment’s superficial muscles—constitute the leg’s “belly”, converging to form the Achilles tendon. This tendon is integral to ankle movement and proper gait [8]. Along with the intrinsic foot muscles, those muscles orchestrate a symphony of movements, from flexion and extension to abduction and adduction of the toes.

The impact of gastrocnemius contracture is profound, silently but mechanically compromising the foot and ankle, leading to disturbed gait and reduced foot energy absorption [9]. The array of treatments spans from symptomatic approaches to conservative stretching regimens and surgical lengthening [7], addressing the altered biomechanics, range of motion, and pain associated with such contractures.

Treatments such as gastrocnemius recession and Baumann procedures have effectively addressed isolated gastrocnemius contracture, ameliorating ankle dorsiflexion [10]. Moreover, gastrocnemius release has proven beneficial for cases unresponsive to conservative treatments, enhancing functional outcomes [11]. Progressive collapsing foot deformity (PCFD) is another pivotal functional cause of forefoot abduction and varus [12].

Despite advancements in treatments, complications like plantarflexion weakness and sural neuritis are not uncommon post-gastrocnemius recession [13]. In this complex landscape, the role of rehabilitation nurses emerges as indispensable. They are the linchpin in enhancing functional outcomes post-surgery and scenarios of limited functional capacity [14]. Their involvement is crucial in early mobilizations, gait sessions, and adaptations, especially when postural changes are prevalent [15].

These interventions are paramount for disorders stemming from gastrocnemius contractures. The importance of rehabilitation nursing extends to crafting strategies for balance and functionality, focusing on strengthening the posterior muscle chains [16]. To our knowledge, and after a preliminary search in Medline (via PubMed) and PROSPERO, there are no recent or undergoing reviews on this topic. In this sense, this paper aims to identify and analyze the existing nursing rehabilitation interventions with a functional goal, following MGSCs, with the aim of preventing foot and ankle disorders.

## 2. Materials and Methods

A narrative review method was conducted, to comprehensively describe, analyze, and interpret relevant sources on the contributions of rehabilitation nursing in addressing medial gastrocnemius silent contractures and preventing foot and ankle disorders.

The study utilized a structured approach, combining contextual analysis of rehabilitation nursing practices with an in-depth exploration of the physiological and clinical characteristics of MGSCs. An interpretative text was formulated, with conclusions derived from the analysis.

Given the novelty of the topic, a narrative review was deemed to be most appropriate. Unlike systematic reviews, which adhere to strict inclusion criteria and predefined methodologies, narrative reviews provide flexibility in exploring diverse sources and perspectives. This approach allows researchers to offer a comprehensive understanding of the subject matter by incorporating a wide range of evidence, including qualitative studies, theoretical frameworks, and expert opinions. Moreover, narrative reviews are particularly beneficial for generating hypotheses, identifying gaps in knowledge, and offering insights into emerging trends or areas requiring further investigation [17].

### 2.1. Eligibility Criteria

The review included those studies focused on functional physical rehabilitation interventions performed by nurses, or with their collaboration, in adult patients (18 years old or more) and in all healthcare contexts (e.g., primary care, clinics, nursing homes, and hospitals). Moreover, only interventions addressing MGSCs were included.

Studies reporting results on pathological MGSCs, namely congenital deformities in pediatric patients (e.g., limb-length discrepancy, cerebral palsy, flatfoot, clubfoot), and secondary conditions, like immobilization for trauma or a nonfunctional limb were excluded.

The search included two types of articles: foundational texts and recent studies (published within the last five years, with priority given to the most current) describing the concept, interventions, and impact of rehabilitation nursing on functional outcomes.

No timeframe restriction was applied, but given the language mastery of the reviewers involved, only Portuguese, English, Spanish, and French studies were included.

### 2.2. Information Sources, Search Strategy and Selection Process

The literature was sourced from a total of four databases and repositories: Medline (via PubMed), CINAHL with full text (via EBSCOHost), Web of Science, and the Cochrane Database of Systematic Reviews, using keywords and subject headings related to medial gastrocnemius silent contractures, rehabilitation nursing, and foot and ankle disorders. The search strategy used for Medline (via PubMed) is presented in Table 1.

## 3. Results

Embarking upon interventions tailored to gastrocnemius contractures necessitates a foundational approach enriched by comprehensive nursing assessments. The first pivotal step involves conducting a discerning examination to differentiate between varying degrees of injuries. This involves thoroughly reviewing the patient’s clinical history and visual inspection, palpation, and functional assessments of joints and muscles [18].

Goniometry is a clinical cornerstone for measuring range of motion (ROM), offering precise and reproducible insights into the extent of impairment and the trajectory of recovery [18]. Evaluating specific movements, such as ankle dorsiflexion, plantar flexion, inversion, and eversion, forms the crux of this comprehensive examination, thus facilitating the formulation of bespoke rehabilitation strategies [18]. It is crucial to evaluate the condition of MGSCs to formulate effective rehabilitation strategies. This evaluation typically involves a thorough clinical history and patient interview to identify potential causes and risk factors, such as prolonged immobilization or repetitive strain. A detailed physical examination, including visual inspection and palpation, helps detect signs of muscle atrophy, tightness, and reduced joint mobility. Goniometry is employed to measure the range of motion (ROM) of the ankle joint, providing critical data on dorsiflexion and plantarflexion. Functional tests, such as the Silfverskiöld test, can help differentiate between gastrocnemius and soleus tightness. Additionally, imaging techniques like ultrasound or MRI may be utilized to assess muscle structure, and patient-reported outcome measures, such as the Foot and Ankle Disability Index (FADI) or the Visual Analog Scale (VAS) for pain, can provide insights into the impact of MGSCs on daily activities and quality of life.

However, the spectrum of assessment extends beyond the physical realm, incorporating a holistic examination of functional abilities, pain thresholds, and psychosocial dynamics. With a patient-centric approach, nursing strategies can be meticulously tailored to meet individual needs and address unique challenges [18].

Following the assessment, the collated information is a bedrock for developing a comprehensive and individualized care plan, amalgamating therapeutic exercises, manual therapies, patient education, and assistive devices to enhance functional outcomes and mitigate complications [18].

### 3.1. Vibration Therapy

Vibration therapy (VT) has garnered considerable attention as a burgeoning modality in rehabilitation nursing, showing promise in addressing gastrocnemius contractures and subsequent foot and ankle disorders [19]. As a non-invasive adjunct to traditional rehabilitation, VT applies controlled vibrations to sensory receptors and muscle spindles, promoting muscle relaxation and improving blood circulation, thereby reducing contractures and associated disorders [20].

Studies suggest VT’s potential to enhance bone density and accelerate fracture healing, presenting a promising avenue for tissue repair and rehabilitation in trauma-induced foot and ankle injuries [21]. VT is a versatile rehabilitation tool with applications ranging from increasing muscle temperature to improving flexibility and reducing stiffness [22,23].

In stroke patients, segmental muscle vibration (SMV) has showcased positive outcomes in enhancing gait speed, stride length, and ankle dorsiflexion [24]. Tailoring VT intensity and incorporating it with stretching and strengthening exercises can contribute to functional improvement and quality of life [25].

In conclusion, integrating VT within the holistic framework of rehabilitation nursing can potentiate synergistic outcomes, amalgamating traditional techniques and patient education to expedite recovery and elevate patient satisfaction. However, uncovering VT’s potential is ongoing, necessitating further research to elucidate its mechanisms and optimize its application in varied disorders.

### 3.2. Stretching-Based Interventions

Stretching-based interventions, fundamental to rehabilitation nursing, offer significant promise in addressing gastrocnemius contractures, foot and ankle disorders, and injuries resulting from trauma. Targeted stretching exercises focusing on the gastrocnemius muscle and surrounding tissues facilitate elongation and improvement of ROM, alleviating contractures and reducing discomfort [26]. When aptly integrated into rehabilitation plans, stretching exercises are a preventative measure against secondary complications such as pressure ulcers and joint deformities [27].

The benefits of stretching-based interventions are particularly noteworthy in the context of trauma-induced foot and ankle injuries. The resultant muscle stiffness and decreased joint flexibility from post-traumatic immobility can hinder functional recovery. Herein, rehabilitation nursing professionals play a pivotal role in implementing specific stretching protocols to overcome these impediments, aiding tissue healing and overall rehabilitation. Additionally, these professionals are instrumental in instructing patients on correct stretching techniques and ensuring adherence to safety protocols, which is crucial for realizing positive outcomes.

For those grappling with diminished lower limb muscle strength and mass, stretching exercises are a viable solution to maintain and enhance ROM. A recent randomized controlled trial (RCT) involving 15 participants underscored the benefits of high-volume stretching exercises on ankle dorsiflexion ROM and muscle stiffness. However, it was noted that these effects diminished post-detraining [28].

Typically, within interventional rehabilitation programs, stretching is incorporated towards the conclusion of sessions, spanning approximately 10 min, and is often paired with respiratory exercises aimed at relaxation.

Focal muscle vibration (FMV) is an alternative stretching technique contributing to alterations in the medial gastrocnemius architecture. This method has positively impacted muscle architecture, including changes in muscle thickness, fiber bundle length, and pennation angle, particularly benefiting individuals with restricted ankle dorsiflexion [9].

### 3.3. Balance

Balance exercise interventions are fundamental to rehabilitation nursing and are pivotal in addressing gastrocnemius contractures, foot and ankle disorders, and trauma-related injuries. By incorporating exercises involving the gastrocnemius muscle, these interventions aim to optimize the neuromuscular system, enhancing strength, coordination, and balance control. As patients experience improved balance, it fosters a sense of confidence in their movements and boosts overall functional independence [29].

Therapeutic balls serve as practical tools in such interventions with unstable surfaces, promoting muscle activation and stabilizing the trunk [30]. A study involving 12 older adults who underwent a virtual rehabilitation program exhibited notable improvements, as reflected in the Berg Balance Scale, accompanied by increased activation of the lateral gastrocnemius muscle [31].

In cases where a nursing diagnosis reveals compromised body balance, a variety of specific nursing interventions can be implemented. These include the application of tactile stimuli with a two-finger touch on the shoulder homolateral to the imbalance; mirror-assisted postural re-education techniques; postural correction; and balance training in various positions, such as lying down, sitting, and standing. Exercises like the hook and bridge, waist dissociation, cross-facilitation, and coordination of body movements are also beneficial [32].

### 3.4. Footwear

The selection and management of footwear stand as integral elements of rehabilitation nursing, particularly when addressing foot and ankle disorders caused by gastrocnemius contractures. Proper footwear can have a profound effect on the course of a patient’s recovery by reducing strain on affected muscles and joints. Shoes that offer appropriate arch support, adequate cushioning, and an optimal fit are essential in reducing pressure on the calf muscles, thereby alleviating the symptoms associated with gastrocnemius contractures. Additionally, footwear designed with rigid heel counters and lateral support can enhance foot stability, helping to prevent ankle sprains and other traumatic injuries, which are common in patients with compromised lower limb function [33,34].

Specialized footwear, such as Masai Barefoot Technology (MBT) shoes, has gained attention for its role in rehabilitation. MBT shoes have been shown to increase muscle activity in the gastrocnemius, especially during the initial phase of walking. This increased activity helps to strengthen the muscle, potentially mitigating the impact of contractures. Studies have also indicated that when MBT shoes are worn in situations of imbalance, the muscle amplitude of both the medial gastrocnemius and the tibialis anterior significantly improves. This enhancement in muscle activation promotes better stability and functional balance during the gait, making MBT shoes a valuable tool in rehabilitation for individuals with muscle contractures or instability [35].

Acknowledging the importance of footwear in alleviating the symptoms of gastrocnemius contractures and preventing further complications allows rehabilitation nurses to play a crucial role in selecting appropriate shoes that support recovery. Rehabilitation nurses must carefully assess each patient’s individual needs to recommend footwear that supports optimal recovery, improves mobility, and reduces the risk of injury. By incorporating shoes that enhance muscle activation and provide stability, such as MBT shoes, nurses can better facilitate the rehabilitation process, ensuring that patients regain functional independence and experience improved overall outcomes.

### 3.5. Objective Assessment of Stiffness

Assessing and evaluating stiffness are paramount in managing foot and ankle disorders, especially when addressing gastrocnemius contractures and related issues. Techniques for objective assessment yield crucial insights into the musculoskeletal condition, aiding rehabilitation nurses in crafting targeted interventions to achieve optimal patient outcomes.

A noteworthy study conducted by Morgan et al. [36] delved into the interplay between the symptomatic Achilles tendon and its influence on the tone and dynamic stiffness of the gastrocnemius muscle. Given its significant role in ankle joint movements and overall gait mechanics, any functional alterations in the Achilles tendon can impart extensive implications on foot and ankle biomechanics.

The study utilized two divergent assessment methods: weight-bearing (WB) and non-weight-bearing (NWB), offering complementary views and facilitating a holistic understanding of the patient’s condition [37].

The WB method assesses muscle tone and dynamic stiffness under the condition of the patient bearing their total body weight. The imposed load during activities such as walking or standing can substantially shape the musculoskeletal system’s response. This is particularly pertinent for patients grappling with foot and ankle disorders, including gastrocnemius contractures, as it sheds light on how these conditions interfere with functional movements [38].

Rehabilitation nurses employ WB assessments to scrutinize the gait patterns, pinpoint abnormalities or compensatory actions, and evaluate the foot and ankle alignment during weight-bearing activities. This insight is invaluable for comprehending the impact of gastrocnemius contracture on weight distribution, joint stability, and muscle activation patterns during daily activities [39].

Moreover, WB assessments are instrumental in identifying gait deviations stemming from gastrocnemius contractures, such as foot drop or abnormal foot pronation. Detecting these irregularities enables nurses to customize interventions, addressing specific gait deficits to re-establish optimal biomechanics.

Conversely, the NWB assessment appraises muscle tone and dynamic stiffness in a position where the patient is not bearing weight, allowing for a more isolated examination of muscle function and stiffness. Typically conducted while the patient is lying or sitting, this approach facilitates effective manipulation and evaluation of the affected foot and ankle [40].

Rehabilitation nurses conduct NWB assessments to measure the passive range of motion (ROM) of the foot and ankle, identifying any limitations or restrictions due to gastrocnemius contractures. This information proves indispensable for determining the degree of contracture and formulating stretching-based interventions tailored to individual needs [41].

Accordingly, NWB assessments yield essential data regarding muscle tone and resistance to passive movement. An increase in muscle tone and dynamic stiffness can signal the presence of gastrocnemius contractures, necessitating specific interventions to relieve tightness and enhance joint mobility.

**Figure 1:**
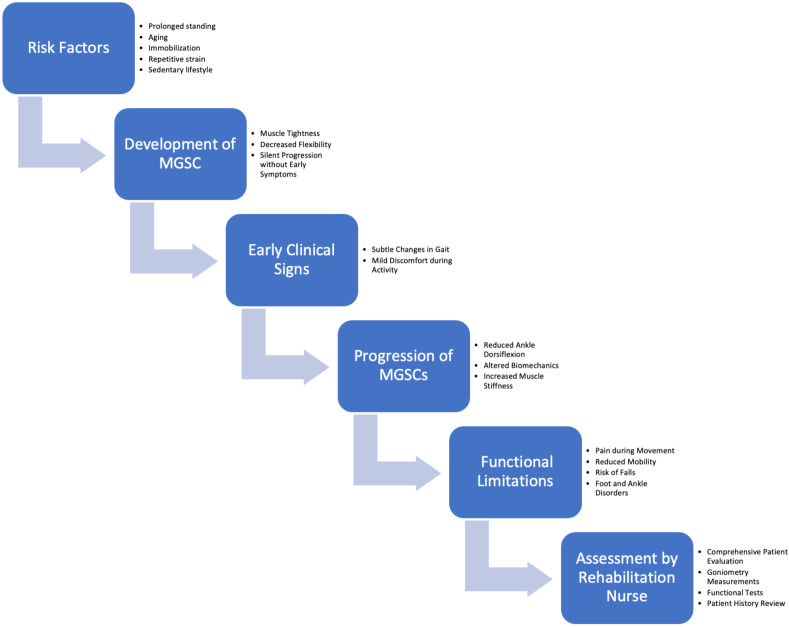
Schematic of the identification of risk factors, the description of clinical disorders, and proposed assessment by rehabilitation nurses.

Figure 1 outlines the progression of medial gastrocnemius silent contractures (MGSCs), beginning with risk factors that lead to the development of MGSCs. This is followed by early clinical signs, which, if untreated, lead to the progression of MGSCs. As the condition worsens, functional limitations arise, affecting mobility and daily activities. The final step is the assessment by a rehabilitation nurse, where the nurse evaluates the patient and develops a tailored rehabilitation plan to address the functional limitations and prevent further complications. This schematic highlights key points where intervention is crucial.

## 4. Discussion

### 4.1. Implications of Functional Interventions

**Figure 2:**
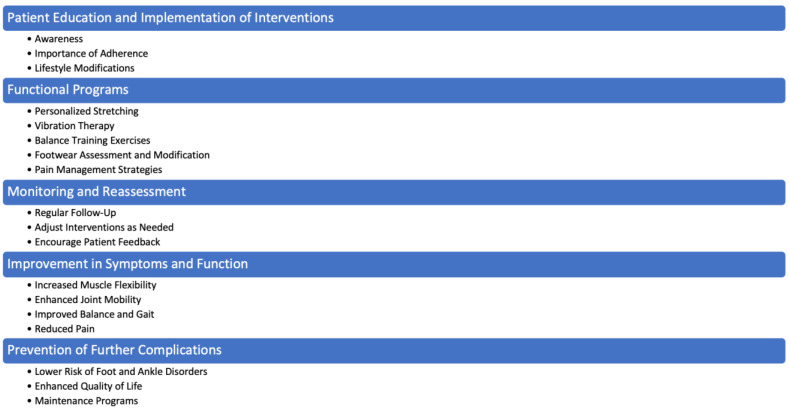
Main intervention points.

The interventions elucidated in this manuscript present promising avenues for addressing muscle-related foot and ankle issues. Figure 2 provides a synthesis of the main intervention points to be addressed in these situations. This includes insights from both foundational texts and recent studies. Foundational texts have provided the theoretical underpinnings and historical perspectives necessary for understanding the pathophysiology of MGSCs and the general principles of rehabilitation nursing. For example, foundational works such as those reviewed early in our methodology were crucial in establishing the basic concepts of gastrocnemius contractures and their impact on foot and ankle biomechanics. Recent studies, such as those included in our results section [19,20,21,22], have built on this foundation by exploring specific interventions like vibration therapy and surgical techniques, providing up-to-date evidence on their effectiveness and clinical application.

Occurrences such as fractures and damage to soft tissue significantly impede the musculoskeletal system, affecting functional status and mobility. In this context, vibration therapy (VT) emerges as a potentially effective and non-invasive supplement to conventional rehabilitation strategies. VT stimulates sensory receptors and muscle spindles, facilitating muscle relaxation, enhancing blood circulation, and promoting neuromuscular activation—factors pivotal for tissue regeneration and functional recovery. Localized vibrations might also expedite the healing of fractures, underscoring the potential advantages of VT in muscle management. Nonetheless, meticulous customization of the frequency and intensity of VT is imperative to cater to individual needs, preventing any adverse effects due to misuse or overexposure [42].

MGSCs can lead to several complications if not appropriately managed. These complications include chronic pain, reduced mobility, and increased risk of falls due to compromised balance and gait abnormalities. Chronic MGSCs can also contribute to the development of compensatory movement patterns, which may cause additional strain and injury to other muscle groups and joints. For instance, patients may experience hip or lower back pain as a result of altered gait mechanics. Furthermore, prolonged immobility and muscle stiffness associated with MGSCs can lead to secondary complications such as pressure ulcers and deep vein thrombosis. Therefore, understanding and addressing these complications is crucial for developing effective rehabilitation strategies and improving patient outcomes.

Stretching-based interventions exhibit considerable potential in managing foot and ankle disorders resulting from muscle-related issues. The immobility following such events often leads to muscle rigidity and decreased joint flexibility, obstructing functional recovery. Rehabilitation nursing professionals possess the expertise to deploy targeted stretching regimes addressing these complications, thereby fostering tissue healing and aiding rehabilitation [43]. However, adapting the timing and intensity of stretching exercises, depending on the healing stage and overall patient condition, is essential [44]. Overstretching or exerting excessive stress on recuperating tissues can be detrimental, potentially compromising the healing trajectory and precipitating further injuries.

Balance exercises emerge as a valuable approach for individuals grappling with muscle-related foot and ankle issues, targeting the neuromuscular system to bolster strength, coordination, and balance control [45]. Enhancing balance can instill confidence in movement and diminish fall risks, a critical consideration during the recuperative phase post-injury. Rehabilitation nurses are responsible for conducting thorough assessments to identify each patient’s distinct balance deficits, subsequently customizing balance exercise programs to maximize their efficacy [46].

Selecting appropriate footwear is a cornerstone in managing foot and ankle disorders and averting complications in muscle-related scenarios. Shoes providing adequate arch support, cushioning, and lateral support, which can alleviate calf muscle pressure, mitigating gastrocnemius contractures [47]. Additionally, appropriate footwear incorporating a rigid heel counter can augment foot stability and curtail the incidence of ankle sprains and related injuries. However, given the diversity in foot shapes and sizes, individualized consideration is paramount when suggesting footwear adaptations [48]. Furthermore, patient education on proper footwear selection and application is indispensable to ensure adherence and optimize therapeutic outcomes.

The practical integration of these interventions into rehabilitation nursing necessitates overcoming potential challenges. Thus, a robust framework that facilitates the seamless adoption of these strategies will be instrumental in realizing their full potential. Such a framework must include clear guidelines for implementation, ongoing training for healthcare professionals, and the establishment of interdisciplinary communication channels to ensure consistent care delivery. Furthermore, nurses and healthcare professionals should consider the accessibility and adaptability of these interventions, taking into account factors such as socioeconomic disparities, patient mobility, and geographical limitations. Ensuring that these interventions are feasible involves making necessary adjustments to the care environment, such as incorporating telehealth for remote consultations or providing access to affordable, specialized equipment. Tailoring interventions to meet each patient’s unique needs and preferences is equally important, as it not only enhances patient adherence but also promotes better outcomes by respecting individual capabilities, goals, and cultural contexts. By focusing on both the practical application and patient-centered customization, healthcare professionals can optimize the effectiveness of these interventions and ensure equitable access to high-quality care.

A patient-centered approach, emphasizing individualized care and extensive patient education, forms the cornerstone of effective rehabilitation nursing. This approach goes beyond simply delivering treatment; it prioritizes understanding and responding to the unique needs, preferences, and values of each patient. By placing the patient at the heart of the rehabilitation process, healthcare professionals can tailor interventions to align with personal goals, cultural considerations, and specific health conditions, thereby ensuring that care is not only clinically effective but also personally meaningful.

This individualized care fosters a sense of autonomy and engagement in patients, as they become active participants in their rehabilitation journey. When patients feel heard and their preferences are incorporated into the treatment plan, their trust in healthcare providers strengthens, which in turn enhances, their commitment to following prescribed regimens. Education plays a crucial role in this process, as well-informed patients are more likely to understand the importance of their treatment, adhere to rehabilitation protocols, and take ownership of their recovery. Comprehensive education about their condition, the rehabilitation process, and self-care strategies empowers patients to manage their health more effectively, reducing the risk of complications or setbacks.

Furthermore, this approach amplifies clinical outcomes by creating a collaborative relationship between patients and healthcare providers. By regularly adjusting care plans based on patient feedback and progress, healthcare professionals can optimize interventions, making them more responsive to changing needs over time. In doing so, rehabilitation nursing not only improves physical recovery but also addresses emotional and psychological well-being, enhancing the overall quality of life. Ultimately, a patient-centered, personalized approach is key to promoting better health outcomes, increasing patient satisfaction, and building long-term trust in the therapeutic process.

### 4.2. The Role of Rehabilitation Nursing in Trauma Care

Rehabilitation nursing plays a central role in muscle care, particularly in cases of trauma-related foot and ankle injuries. The contributions of rehabilitation nurses significantly influence patient outcomes, particularly through the development and implementation of comprehensive, patient-centered care plans [49]. As integral members of the interdisciplinary care team, rehabilitation nurses collaborate closely with physical therapists, orthopedic surgeons, podiatrists, and other healthcare professionals to optimize treatment strategies and outcomes [50]. Their specialized training in musculoskeletal evaluation, therapeutic interventions, and patient education enables them to address the unique needs of patients recovering from muscle-related injuries, including MGSCs and related foot and ankle disorders.

A fundamental aspect of rehabilitation nursing in trauma care is its patient-centered approach. Rehabilitation nurses work closely with trauma teams to ensure the smooth transition from acute care to long-term recovery. This collaboration typically begins immediately after the injury, with trauma team members providing initial emergency care and stabilization. Once stabilized, rehabilitation nurses step in to assess the patient’s functional capacity and long-term recovery needs, which include evaluating musculoskeletal health, mobility, and potential risks for complications. This seamless coordination ensures that rehabilitation begins early and that all aspects of the patient’s condition are addressed promptly, fostering better long-term outcomes.

In the course of patient care, rehabilitation nurses perform intricate musculoskeletal assessments, which provide crucial insights into the patient’s physical condition, level of impairment, and specific recovery needs [51,52]. These assessments go beyond physical examinations and take into account the patient’s lifestyle, personal goals, and emotional well-being, resulting in highly individualized rehabilitation programs. This personalized care not only aims for optimal recovery but also enhances patient satisfaction and quality of life. Rehabilitation nurses also play a key role in educating patients and their families about the injury, the rehabilitation process, and self-care strategies that can be employed at home to continue progress post-discharge. By empowering patients with knowledge and fostering a sense of ownership over their rehabilitation, nurses increase the likelihood of adherence to treatment plans and, thus, improve overall outcomes.

The integration of emerging technologies into rehabilitation nursing is a growing trend that holds great potential for enhancing patient care. Technological tools such as wearable devices, telehealth platforms, and advanced patient monitoring systems offer personalized insights into a patient’s rehabilitation progress. Wearable devices, for example, can track mobility, gait, and muscle function in real-time, providing valuable feedback for both patients and healthcare providers. Telehealth platforms allow for continuous patient support and guidance, particularly for those who may face difficulties accessing in-person care due to geographic or mobility constraints. These technologies allow rehabilitation nurses to make timely adjustments to care plans based on the real-time data gathered, thereby optimizing recovery outcomes and improving patient engagement. Informatics systems also play a role in improving care coordination within interdisciplinary teams, as they allow for better data sharing and collaboration among healthcare providers. This ensures that all members of the healthcare team are fully informed of the patient’s progress, facilitating more cohesive and comprehensive care.

Ethical considerations are an inherent part of rehabilitation nursing practice, particularly in trauma care settings. Rehabilitation nurses must navigate various ethical challenges, such as respecting patient autonomy, ensuring informed consent, and addressing potential inequities in access to care. For example, some patients may have limited access to rehabilitation services due to socioeconomic factors, while others may struggle with the emotional and psychological aspects of recovery. Rehabilitation nurses must advocate for their patients, ensuring that care is equitable, accessible, and aligned with each patient’s values and preferences. Respecting patient autonomy is particularly important in rehabilitation, as patients are often involved in making decisions about their treatment options and recovery strategies. By fostering open communication and building trust, nurses help ensure that patients feel respected and supported throughout their rehabilitation journey.

Professional development is also a key component of effective rehabilitation nursing. Given the dynamic nature of healthcare and the ongoing advancements in musculoskeletal care and rehabilitation, it is critical for rehabilitation nurses to stay current with evidence-based practices and technological innovations. This commitment to lifelong learning allows nurses to provide the most up-to-date, effective care for their patients. Continuing education, certifications in specialized areas of rehabilitation, and participation in research initiatives all contribute to the professional growth of rehabilitation nurses, equipping them with the knowledge and skills needed to address evolving patient needs and challenges.

Adopting evidence-based practices is a cornerstone of rehabilitation nursing, ensuring that interventions are safe, effective, and adaptable to the diverse needs of patients [50]. This approach is supported by ongoing research and clinical trials, which inform the development of new therapeutic techniques and rehabilitation strategies. By utilizing standardized outcome measures, rehabilitation nurses can objectively evaluate the effectiveness of interventions, allowing them to refine rehabilitation programs as needed and contribute to the advancement of the field. These outcome measures not only guide clinical practice but also pave the way for future research, helping to improve patient care and outcomes in the long term.

In addition to clinical expertise, rehabilitation nurses must possess strong communication and leadership skills to effectively coordinate care across interdisciplinary teams. Trauma care often involves multiple healthcare providers working together to ensure the best possible outcomes for the patient. Rehabilitation nurses frequently act as liaisons between the patient, their family, and the wider healthcare team, ensuring that everyone is aligned in their approach to care. Clear and effective communication is essential for fostering teamwork, ensuring that care plans are followed, and facilitating smooth transitions between different stages of care.

Rehabilitation nurses frequently act as liaisons between the patient, their family, and the wider healthcare team, ensuring that everyone is aligned in their approach to care. Clear and effective communication is essential for fostering teamwork, ensuring that care plans are followed, and facilitating smooth transitions between different stages of care. However, despite these critical contributions, challenges remain in optimizing rehabilitation outcomes. In particular, the complexity of managing muscle-related foot and ankle issues, such as MGSCs, requires not only interdisciplinary coordination but also continuous adaptation of care strategies to meet individual patient needs. The evolving nature of healthcare, coupled with the unique characteristics of each patient, introduces complexities that rehabilitation nurses must navigate, highlighting the need for future research and innovation in this area. In addressing these challenges, it becomes crucial to explore new directions, such as integrating emerging technologies, refining evidence-based practices, and developing personalized rehabilitation plans that account for diverse patient profiles and changing clinical contexts.

### 4.3. Challenges and Future Directions

While the interventions discussed in this review present promising opportunities for managing muscle-related foot and ankle issues, they also introduce several challenges that require further exploration. One of the primary difficulties lies in recognizing the diverse range of patient characteristics—such as age, comorbid conditions, activity levels, and overall health—which can significantly influence both the course of treatment and the patient’s response to various interventions. Older adults with reduced muscle strength may respond differently to stretching or balance exercises compared to younger individuals with fewer health complications. Similarly, patients with conditions like diabetes or arthritis may have slower recovery rates, requiring tailored interventions. Rehabilitation nurses must therefore adopt a highly personalized and adaptable approach, adjusting rehabilitation strategies to meet the evolving needs of each patient. Flexibility in modifying interventions is crucial, as it ensures that care is aligned with the patient’s progress and response to treatment over time.

Another challenge is determining the ideal timing, intensity, and duration of each intervention. Rehabilitation for muscle-related injuries, as in the case of MGSCs, requires a careful balance between promoting tissue healing and preventing the application of excessive strain that could delay recovery or cause additional injuries. For example, while stretching-based interventions are highly effective in enhancing muscle flexibility, overstretching can lead to muscle tears or joint damage. Rehabilitation nurses play a pivotal role in continuously monitoring and assessing patient progress to ensure that interventions are adjusted appropriately. Diligent observation and the use of objective outcome measures are essential in identifying when to escalate or taper off certain treatments. This proactive and dynamic approach is critical, given the varied healing trajectories seen in muscle-related injuries, where some patients may recover quickly while others face prolonged rehabilitation periods.

The efficacy of rehabilitation nursing interventions—such as vibration therapy, targeted stretching, balance training, and appropriate footwear—has been well-documented in improving patient outcomes for foot and ankle disorders. However, these interventions are not without their limitations. For instance, vibration therapy may not be suitable for patients with certain cardiovascular conditions, and not all patients may have access to specialized footwear that could prevent complications such as pressure sores or further musculoskeletal strain. Rehabilitation nurses must be aware of these potential drawbacks and ensure that interventions are selected and implemented with caution, taking into account the patient’s broader health profile.

Interdisciplinary collaboration remains a fundamental aspect of managing muscle-related foot and ankle disorders effectively. Rehabilitation nurses work alongside physical therapists, orthopedic surgeons, podiatrists, and other healthcare professionals to deliver a coordinated and comprehensive care plan. This collaboration ensures that all aspects of the patient’s condition are addressed, from surgical interventions to functional rehabilitation. Additionally, educating patients about proper footwear selection, modifications, and fall prevention strategies is critical in preventing reinjury and improving functional outcomes. Rehabilitation nurses must also integrate ongoing evaluation of intervention effectiveness to guarantee that care remains relevant and beneficial throughout the rehabilitation process.

The findings of this review underscore the significant implications for clinical practice. The interventions explored can be seamlessly integrated into standard rehabilitation protocols for patients dealing with foot and ankle disorders, providing rehabilitation nurses with a solid foundation to enhance patient outcomes. However, future research is essential in addressing several gaps in knowledge. For example, more studies are needed to explore the efficacy of specific interventions, such as vibration therapy, and to determine the most effective combinations of these therapies for various patient populations. Investigating the optimal timing and duration of rehabilitation programs will also be critical in refining care protocols.

Technological innovations present exciting opportunities for advancing rehabilitation nursing practice. Emerging tools, such as virtual reality, augmented reality, and telemedicine platforms, could revolutionize the way rehabilitation services are delivered. For example, virtual reality could be used to simulate real-life environments, helping patients practice balance and mobility exercises in a controlled setting. Telemedicine can facilitate remote monitoring and personalized adjustments to care plans, especially for patients in rural or underserved areas who may have limited access to in-person rehabilitation services. Wearable technology could also provide real-time data on muscle activity and joint mobility, allowing rehabilitation nurses to fine-tune interventions based on objective metrics.

While the interventions currently employed in rehabilitation nursing for muscle-related foot and ankle disorders are promising, there are still significant challenges to overcome. Addressing the diversity of patient characteristics, fine-tuning intervention timing and intensity, and navigating the limitations of certain treatments are key areas where rehabilitation nurses must exercise both caution and creativity. Interdisciplinary collaboration and patient education remain central to the success of rehabilitation programs. Moving forward, research should continue to explore novel technologies and refine evidence-based practices, ensuring that rehabilitation nursing remains adaptable, patient-centered, and effective in improving outcomes for individuals with muscle-related injuries. These advancements will not only enhance the quality of care but also foster new pathways for innovation in rehabilitation nursing practice.

## 5. Conclusions

Muscle-focused nursing interventions positively impact the management of foot and ankle disorders, with interdisciplinary collaboration and the involvement of qualified professionals proving crucial. Our findings have substantial implications for clinical practice and pinpoint several avenues for future research. Including proper education on muscle-adapted footwear selection and modification, fall prevention strategies, and continuous monitoring and evaluation of intervention effectiveness is paramount in muscle-focused rehabilitation plans to guarantee optimal outcomes. This study underscores the indispensability of muscle-focused nursing interventions in ameliorating patient outcomes and accentuates the necessity for a thorough and interdisciplinary approach to managing foot and ankle disorders.

In exploring deeper the relationship between various interventions, muscle-focused nursing, and musculoskeletal conditions such as gastrocnemius contractures and muscle-related injuries, we unveil the criticality of this nexus in securing positive patient outcomes. The significance of stretching-based interventions, balance exercises, and judicious footwear selection is fundamental to comprehensive muscle-focused nursing, contributing to enhanced muscle flexibility, joint mobility, postural stability, and overall functional independence.

With its patient-centered and evidence-based approach, muscle-focused nursing is pivotal in catering to the diverse needs of this patient demographic. Through meticulous assessments, crafting personalized care plans, and guiding patients through pertinent interventions, muscle-focused nurses empower patients to actively engage in their recovery journey, thereby fostering an enhanced quality of life.

For muscle care, the implications of the discussed interventions are notably substantial. Muscle-related injuries can significantly impede an individual’s mobility and functionality. Integrating stretching-based interventions and balance exercises is instrumental in reinstating muscle flexibility, joint stability, and proprioceptive awareness. Education focusing on appropriate muscle-adapted footwear selection emerges as a crucial determinant in circumventing secondary complications and augmenting the healing trajectory for muscle-induced injuries. The amalgamation of interdisciplinary expertise involving muscle-focused nurses, orthopedic specialists, and physical therapists is vital in optimizing muscle care management, ensuring holistic and customized interventions culminating in successful recoveries and enriched patient well-being. Recognizing the multifactorial nature of MGSCs, the identified evidence underscores the efficacy of targeted stretching exercises, vibration therapy, and balance training in treating MGSCs arising from diverse causes such as prolonged immobilization, repetitive strain, or underlying neuromuscular disorders. This focus on etiology-based intervention strategies reinforces the clinical relevance of our study and provides a clearer guide for practitioners in selecting the most appropriate therapeutic approaches to enhance functional recovery and quality of life for patients with MGSCs.

Moreover, technological advancements and digital health tools are poised to revolutionize muscle-focused nursing, offering innovative solutions for personalized care and real-time monitoring. Future research should explore the integration and efficacy of such technologies in enhancing muscle-focused rehabilitation strategies and patient outcomes.

Acknowledging any limitations of the current study while not undermining the findings also fosters a balanced perspective. This candid approach encourages further exploration and examination in the field, driving the continual evolution of knowledge and practice in muscle-focused nursing.

Recognizing the complex interplay between muscle-focused nursing, targeted interventions, and the unique challenges presented by various musculoskeletal conditions is essential to advancing this field. By acknowledging any limitations of the current study without detracting from its core findings, a balanced perspective is maintained, which supports further exploration and examination. This open and candid approach helps drive the continual evolution of knowledge and practice in muscle-focused nursing. Ongoing research, fostering collaboration across healthcare disciplines, and a steadfast commitment to patient-centered care are crucial components in refining and advancing rehabilitation strategies. Such efforts are vital for achieving superior functional outcomes and, ultimately, improving the quality of life for individuals affected by musculoskeletal conditions.

## Figures and Tables

**Table 1:** Search conducted in Medline (PubMed).

Search (Query)	Records Retrieved
((((Foot Disorders [Title/Abstract] OR Ankle Disorders [Title/Abstract] OR Gastrocnemius Contracture [Title/Abstract] OR Silent Contractures [Title/Abstract]) OR (Achilles Tendon [MeSH] OR Gait Disorders, Neurologic [MeSH] OR Postural Balance [MeSH])) AND (Rehabilitation Nursing [Title/Abstract] OR Functional Recovery [Title/Abstract] OR Post-Surgery Rehabilitation [Title/Abstract] OR Range of Motion [Title/Abstract] OR Muscle Strength [Title/Abstract])) OR Rehabilitation Nursing [MeSH]) Filters: in the last 5 years, English, Portuguese, Spanish, MEDLINE	983

## Data Availability

All data were reported in the review manuscript.
